# Quality of working life of employees in public healthcare organization in Finland: A cross‐sectional study

**DOI:** 10.1002/nop2.1896

**Published:** 2023-06-19

**Authors:** Reetta Kesti, Outi Kanste, Jenni Konttila, Anne Oikarinen

**Affiliations:** ^1^ Master of Health Sciences, Research Unit of Nursing Science and Health Management, Faculty of Medicine University of Oulu Oulu Finland; ^2^ PhD, Adjunct Professor, University Lecturer, Research Unit of Nursing Science and Health Management, Faculty of Medicine University of Oulu Oulu Finland; ^3^ PhD, Post‐doctoral Researcher, Research Unit of Nursing Science and Health Management, Faculty of Medicine University of Oulu Oulu Finland; ^4^ PhD, University Lecturer, Research Unit of Nursing Science and Health Management, Faculty of Medicine University of Oulu Oulu Finland

**Keywords:** employees, healthcare professionals, management, public sector, quality of working life

## Abstract

**Aim:**

Work dissatisfaction, burnout and workforce shortage are major problems in healthcare globally, all of which are associated with QWL. Previous studies have found that the QWL of healthcare professionals is moderate or low. The aim was to describe the quality of working life (QWL) of employees working in public healthcare and describe the association between QWL and background variables.

**Design:**

This study had a quantitative cross‐sectional survey design.

**Methods:**

Data was collected from the employees of a Finnish healthcare organization in autumn 2021 using an online questionnaire (*n* = 837). Convenience sampling was used in the selection of the healthcare organization. The study was reported according to STROBE guidelines.

**Results:**

The QWL was moderate, and the mean QWL index calculated from the questionnaire responses was 0.524. The QWL index was lowest in healthcare professionals and highest in upper management, with some dissatisfaction towards leadership noted.

## 1 INTRODUCTION

The 2030 Agenda for Sustainable Development includes goals to promote sustainable economic growth and productive employment to ensure healthy lives and well‐being for all (United Nations, 2015). According to the World Health Organization, to reach the goal of health and well‐being, the world needs 9 million more nurses and midwives by 2030. Indeed, there is a lack of healthcare workforce globally, particularly nurses and midwives, who represent almost 50% of all healthcare employees. (WHO, 2020) Finland has reported dissatisfaction among healthcare professionals and many Finnish unions organized strikes during spring 2022, demanding better rewards and working conditions (STTK, 2022).

The global shortage of nurses is threatening healthcare systems and the well‐being of healthcare employees. One reason for the nurse shortage, in addition to increased care needs, is that organizations have difficulties retaining nurses due to job dissatisfaction. (Scammell, 2016) The coronavirus disease (COVID‐19) pandemic has affected healthcare professionals and caused mental distress (Braquehais et al., 2020) and other psychological effects such as depression, anxiety and insomnia (De Kock et al., 2021). Moreover, the fear of COVID‐19 during the pandemic has decreased job satisfaction and increased turnover intentions of nurses (Labrague & Santos, 2020). The pandemic has put healthcare employees under considerable pressure, and the labour shortage is estimated to worsen during the crisis in Finland. Additionally, there are service backlogs and long queues in healthcare, coupled with more people requiring support due to the pandemic (Labrague & de los Santos, 2021).

Burnout can increase nurses' intentions to leave their profession, while satisfaction with the work environment decreases it; this includes satisfaction with staffing levels, leadership, quality of care, relationships and participation in hospital affairs (Sasso et al., 2019). Nurses in Finland have expressed uncertainty towards the work culture and environment, which can increase their intentions to leave. This is also affected by education, age and work shifts, which further impact how nurses feel about management and how empowered they feel themselves. (Hahtela et al., 2015) Fairness in interpersonal relations with managers and justice in decision‐making can increase job satisfaction and reduce intentions to leave (Zahednezhad et al., 2021).

A transformational leadership style, which can be described as inspiring and motivating leadership, has been found to have a positive effect on predicted nurse turnover (Suliman et al., 2020). Transformational leadership styles can improve the work environment and have a positive influence on workforce and organizational outcomes, such as burnout, job satisfaction, nurse retention and productivity. Managerial competence can also affect employees' organizational commitment. (Almutairi & Bahari, 2022.) In contrast, task‐focussed leadership styles can have a more negative effect on workforce outcomes (Cummings et al., 2018).

Quality of working life (QWL) is a multidimensional concept that usually refers to an individual's feelings or attitude towards the work organization and job and includes aspects such as opportunity to develop, opportunities to utilize one's talents, compensation, impact on personal life and well‐being at work. QWL is connected to job satisfaction and perception of fairness in organization's operating culture. (Totawar & Nambudiri, 2014.) However, as QWL has many definitions and no clear consensus of the concept, there remains a lack of standard method to measure QWL (de Lira et al., 2021). It is crucial to improve QWL and organizational performance simultaneously. In the context of healthcare, there is a need to enhance labour productivity due to the workforce shortage. (Pot & Koningsveld, 2009.) To enhance effectiveness in healthcare activities, implementing measures to address staff shortages and improving the competence of head nurses in human resource management and planning can be beneficial (Nobakht et al., 2018).

**FIGURE 1:**
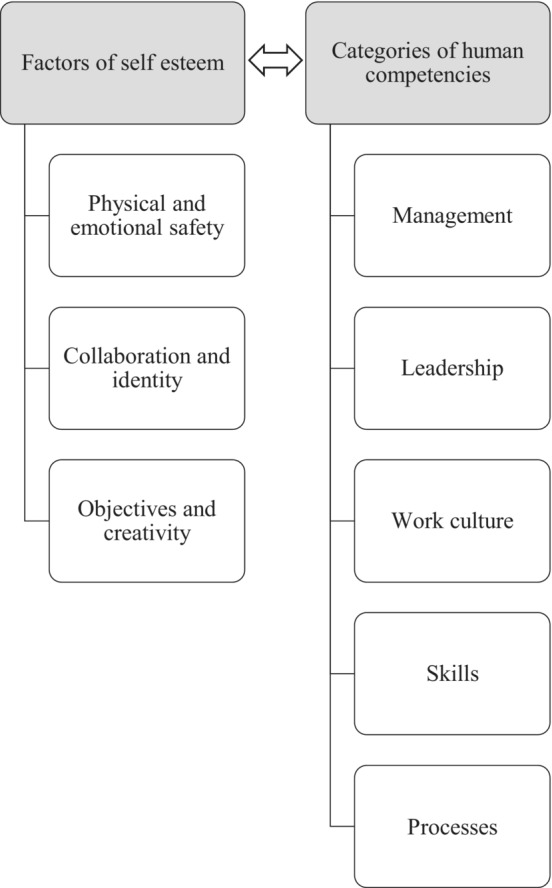
Conceptual framework of the study.

In this study, we employed a method outlined by Kesti et al. (2016), in which QWL is seen as a human intangible factor of production, the development of which can promote labour productivity. Kesti et al. introduced factors of self‐esteem and categories of human competencies as factors influencing QWL (Figure 1). Human competencies represent the knowledge‐based success factors of an organization and are included in measuring the factors of self‐esteem. Physical and emotional safety is the foundation of a good QWL, while excessive stress and fear have a negative effect on employee well‐being and consequently, productivity. The collaboration and identity factor is mostly linked to group motivation, while the objectives and creativity factor refers to the positive attitude and enthusiasm of employees (Kesti et al., 2016).

The QWL of nurses is affected by factors such as age, job nature, level of education, sleep disorders and adverse care events (Wang et al., 2020). Human resource management has a significant role in improving the QWL of nurses (Raeissi et al., 2019; Wang et al., 2020). Job burnout has become increasingly common among nurses and is negatively correlated with QWL (Wang et al., 2019). Moreover, anxiety related to COVID‐19 has been shown to be associated with lower QWL (Aydın et al., 2021). Occupational stress is related to QWL, and managers play a key role in preventing occupational stress through modifying the work environment; this can be achieved by providing opportunities for skill development, enhancing work conditions, developing functions and offering psychological support (de Lira et al., 2021). Improvement of the QWL has been shown to positively impact workforce retention and psychological well‐being (Hardjanti et al., 2017). QWL is related to organizational commitment. To increase QWL and organizational commitment, the emphasis should be on providing opportunities for skill development, ensuring job security and enhancing employee participation in decision‐making (Aminizadeh et al., 2022).

The workforce shortage, work dissatisfaction and job burnout are challenges in healthcare globally, and in Finland. The Finnish government is committed to ensuring access to quality healthcare services for all, while restraining the growth of costs. This is to be achieved with the health and social services reform from the year 2023 onwards. One aspect of this reform is to improve employees' well‐being, develop management and support knowledge‐based management. (Finnish Government, 2022) QWL is less studied in Finnish healthcare context. This study aims to describe the QWL of employees working in public healthcare in Finland. According to the theory basis of this study, QWL is related to many organizational outcomes, stress, burnout, organizational commitment and job satisfaction; thus, it is beneficial to investigate the current status of QWL. QWL has been mainly studied among nurses, but we also examine QWL in relation to other professionals' and managers' viewpoint. Describing these aspects helps in recognizing the problems and potential for development in healthcare. It also enables the focus on knowledge‐based management to enhance the quality of work life (QWL) within the healthcare setting.

## 2 THE STUDY

### 2.1 Aims and objective

The aim of this study was to describe the QWL of employees working in public healthcare and to describe the association between the QWL and background variables. The research questions were as follows:

1. What are the QWL and its factors of employees working in public healthcare?
2. What is the association between the background variables and the qwl of employees working in public healthcare?

## 3 METHODS

### 3.1 Design

This study had a quantitative cross‐sectional survey design (Polit & Beck, 2017), where data were collected with a self‐reported online questionnaire. The STROBE checklist for cross‐sectional studies was used as a guideline for reporting (von Elm et al., 2007).

### 3.2 Population and sample

The data were collected from a Finnish public healthcare organization in one of the 21 hospital districts in Finland. The district includes an emergency hospital with 247 hospital beds and about 2000 employees. Convenience sampling was used to select the hospital district (Polit & Beck, 2017). In Finland, public healthcare services include primary and special healthcare, which are provided by municipalities and hospital districts. At the time of data collection, the hospital districts were responsible for arranging special medical care in their area; due to reform of the healthcare services in Finland, from 2023 onwards, their responsibility will transfer to new well‐being services counties (Finnish Government, 2022).

### 3.3 Data collection

The data were collected in autumn 2021 using an online questionnaire that was sent to all employees in the hospital district via e‐mail (*n* = 2077). The study participants were contacted by the organization's human resource professional. The data were collected by the hospital district. A total of 837 employees responded to the questionnaire, with a response rate of 40%. The study participants included nurses and other healthcare professionals, other workers, physicians and managers (first‐line, middle and top management).

### 3.4 Instrument

#### 3.4.1 Measuring personal and work‐related characteristics

Personal and work‐related characteristics of the study participants were measured using three questions that gathered information about age, job title and managerial position.

#### 3.4.2 QWL questionnaire

QWL was measured with a questionnaire and method of analysis developed by Kesti et al. (2016). The questionnaire consists of 15 questions to measure perceptions in leadership, management, work culture, skills and work processes (Table 1). The answers were given on a 5‐point Likert scale with the following ratings: (1). strongly disagree, (2). disagree, (3). neither agree nor disagree, (4). agree and (5). strongly agree.

**TABLE 1:** Questions in the quality of working life questionnaire sorted by factors of self‐esteem and measured Cronbach's alpha values.

Questions measuring factors of self‐esteem	Cronbach's alpha
Physical and emotional safety (PE)	0.66 (*n* = 801)
1. Top management shows that it cares about the well‐being of employees at work	
2. I find the actions of my line manager to be impartial and fair	
3. In my own work community, I can safely share my own views and experiences	
4. I feel that my skills are sufficient to perform my job effectively	
5. We operate according to jointly agreed practices	
Collaboration and identity (CI)	0.74 (*n* = 791)
1. Upper management communicates openly and interactively	
2. My first‐line manager promotes and supports interprofessional collaboration in our organization	
3. We work seamlessly in interprofessional collaboration for the benefit of our customers	
4. We can use our expertise to improve workflow and the customer experience	
5. The tasks and goals are sufficiently clear, such that I understand what is expected of me	
Objectives and creativity (OC)	0.77 (*n* = 765)
1. Upper management leads us so that we are well prepared for the future	
2. My first‐line manager inspires and encourages me to develop ways of working	
3. We help each other to succeed	
4. My job offers me the opportunity for professional development	
5. We are constantly developing our operations, taking advantage of the feedback we receive from our customers and the opportunities offered by digitalization	

The questions incorporated the following factors of self‐esteem: physical and emotional safety (PE), collaboration and identity (CI), and objectives and creativity (OC; Table 1). Kesti et al. argued that the impact of intangible assets on organizational performance is connected to the situational combinations of these factors and that each of these factors has a different effect on performance. The QWL index can be calculated by considering the different effects of the factors of self‐esteem. The index is always between 0% and 100%; therefore, the QWL index represents the utilization percentage of intangible assets. Kesti et al. based this calculation on different motivational theories, such as those outlined by Hertzberg (1959) and Kano et al. (1984). The QWL index is calculated using the following equation:

$$\mathrm{QWL}=\mathrm{PE}\left(x\_1\right)\times \left(\left(\mathrm{CI}\left(x\_2\right)+\mathrm{OC}\left(x\_3\right)\right)÷2\right)$$

where PE(x_1), CI(x_2) and OC(x_3) are the coefficients describing motivation theoretical effects in human performance, and the x‐values are the outcomes of each self‐esteem factor calculated from the survey results (Kesti et al., 2016).

The methodology of measuring QWL through factors of self‐esteem and calculating the QWL index has been proven to be reliable (Kesti et al., 2016). The Cronbach's alpha values for the factors of self‐esteem were 0.66, 0.74 and 0.77, where an alpha value of ≥0.70 is considered acceptable and ≥0.80 is desirable (Polit & Beck, 2017). The questionnaire is less studied in the healthcare context; thus, we recommend further development of the questionnaire.

### 3.5 Data analysis

The data were analysed using IBM SPSS Statistics 27. The descriptive data are presented as frequencies and percentages. The missing values were not replaced. The QWL index could be calculated for 99% (*n* = 829) of the participants due to high rates of missing data in the remaining 1% of participants. The QWL index is presented as the mean, 95% confidence interval (CI) and standard deviation (SD). The assumption of normality of distributions was estimated from histograms and tested by the Kolmogorov–Smirnov test. Parametric tests were used because the QWL index was close to normal distribution in all groups. In some cases, nonparametric tests were used due to finding more skewness when analysing the sum variables. The connection of age and job title to QWL index was tested with one‐way ANOVA and post hoc test Tukey's test, while the connection of managerial position was tested using *t*‐test. The differences in the mean scores of the sum variables between job title groups and age groups were tested by one‐way ANOVA or Kruskal–Wallis test, and the managerial position was tested by *t*‐test or Mann–Whitney test. The internal consistency of the sum variables was estimated with Cronbach's alpha.

### 3.6 Ethical considerations

The study was conducted considering the essential research ethics of the Declaration of Helsinki (WMA, 2013) and following the ethical guidelines of the Finnish Advisory Board on Research Integrity (TENK, 2019). In Finland, an ethical review statement is only needed for studies with certain research designs. In this study, an ethical review statement from the Human Sciences Committee was not required (TENK, 2019). Research permission was given by the organization. The data were sent to the researcher in anonymized form and were handled in such a way that no respondent could be identified. Responding to the questionnaire was voluntary, and all participants provided informed consent.

## 4 RESULTS

### 4.1 Personal and work‐related characteristics

Most of the participants were between 30 and 59 years old (Table 2). The largest age group was 50–59 years old, which included 28% of the respondents, and the smallest age group was ≥60 years, with 9.8% of the respondents. A total of 42 study participants chose not to disclose their age. More than half of the participants were healthcare professionals, and approximately one‐third were specialists or other workers. Only 3.9% of the participants were physicians, 4.4% were working as first‐line managers, and 3.0% were in leading upper management; among all participants, 7.4% were in a managerial position.

**TABLE 2:** Descriptive data and QWL index according to age, job title and managerial position.

Personal characteristics	% (*n*)	QWL index (*n* = 829)				
		*M*	*p* ^a^	95% CI		SD
				Lower	Upper	
Age (years)						
<30	13.2 (104)	0.520	0.094	0.491	0.549	0.150
30–39	25.1 (197)	0.505		0.486	0.525	0.138
40–49	23.8 (187)	0.524		0.500	0.547	0.165
50–59	28.0 (220)	0.529		0.510	0.549	0.148
≥60	9.8 (77)	0.561		0.525	0.596	0.157
Job title						
Healthcare professional 1	54.3 (446)	0.496	<0.001	0.483	0.510	0.145
Specialist or other worker 2	34.4 (283)	0.539		0.521	0.557	0.152
Physician	3.9 (32)	0.575		0.523	0.627	0.144
First‐line manager 3	4.4 (36)	0.618		0.579	0.657	0.115
Leading upper management 4	3.0 (25)	0.652		0.592	0.712	0.145
Manager position						
Yes	7.4 (61)	0.632	<0.001	0.599	0.665	0.128
No	92.6 (767)	0.515		0.505	0.526	0.150

*Note*: 1 Registered nurse, licensed practical nurse, midwife, paramedic, physiotherapist, occupational therapist and other. 2 For example, employees in hospital supportive services. 2For example, employees in hospital supportive services. 3 Head nurse, department manager or service manager. 4 Area director, unit director, nursing supervisor, member of hospital district executive team.

Abbreviations: CI, confidence interval; *M*, Mean; QWL, quality of working life; SD, standard deviation.

^a^ One‐way ANOVA/*t*‐test.

### 4.2 QWL

#### 4.2.1 QWL and connection to background variables

From the total number (*n* = 837) of participants, the QWL index could be calculated for 99% (*n* = 829) of the participants (Table 2). The mean QWL index was 0.524 (SD: 0.15; 95% CI: 0.514, 0.534). The minimum and maximum QWL index was 0.126 and 1.00, respectively. The mean QWL was at a moderate level, and the range of QWL indices was high throughout the whole organization.

Between job title groups, the mean QWL index varied significantly between 0.496 and 0.652 (*p* < 0.001; Table 2). The QWL index was lowest in healthcare professionals and highest in leading upper management. Closer inspection with Tukey's test showed a significant difference in the QWL index between healthcare professionals and all other job titles (Table 3). The QWL index was higher in participants working in a managerial position compared to those who did not (*p* < 0.001). When comparing age groups, the QWL index was the highest (0.561) in workers aged ≥60 years, and it was 3%–6% lower in younger age groups, albeit with no statistically significant difference (*p* = 0.094).

**TABLE 3:** Pair comparison of the QWL index between job title groups with Tukey's test.

Job title (A)	Job title (B)	MD (A‐B)	SE	*p*	95% CI	
					Lower	Upper
Healthcare professional	Specialist or other worker	−0.042	0.011	0.001^a^	−0.073	−0.012
	Physician	−0.079	0.027	0.028^a^	−0.152	−0.006
	First‐line manager	−0.121	0.025	0.000^a^	−0.191	−0.052
	Leading upper management	−0.156	0.030	0.000^a^	−0.238	−0.073
Specialist or other worker	Healthcare professional	0.042	0.011	0.001^a^	0.012	0.073
	Physician	−0.036	0.027	0.671	−0.111	0.038
	First‐line manager	−0.079	0.026	0.020^a^	−0.150	−0.008
	Leading upper management	−0.113	0.031	0.002^a^	−0.197	−0.030
Physician	Healthcare professional	0.079	0.027	0.028^a^	0.006	0.152
	Specialist or other worker	0.036	0.027	0.671	−0.038	0.111
	First‐line manager	−0.043	0.036	0.754	−0.140	0.055
	Leading upper management	−0.077	0.039	0.283	−0.184	0.030
First‐line manager	Healthcare professional	0.121	0.025	0.000^a^	0.052	0.191
	Specialist or other worker	0.079	0.026	0.020^a^	0.008	0.150
	Physician	0.043	0.036	0.754	−0.055	0.140
	Leading upper management	−0.034	0.038	0.896	−0.139	0.070
Leading upper management	Healthcare professional	0.156	0.030	0.000^a^	0.073	0.238
	Specialist or other worker	0.113	0.031	0.002^a^	0.030	0.197
	Physician	0.077	0.039	0.283	−0.030	0.184
	First‐line manager	0.034	0.038	0.896	−0.070	0.139

Abbreviations: CI, Confidence interval; MD, mean difference, SE, standard error.

^a^ Statistically significant.

#### 4.2.2 Factors of self‐esteem and association with background variables

Physical and emotional safety (PE) was the highest evaluated factor of self‐esteem (mean [M]: 3.397), with Collaboration and identity (CI) at a similar level (M: 3.345; Table 4). Objectives and creativity (OC) were the lowest evaluated factor (M: 3.137). All factors of self‐esteem were the lowest in healthcare professionals compared with other job title groups (*p* < 0.001; Table 4). Respondents who were higher in the hierarchy tended to award higher scores. Leading upper management evaluated PE and CI as being relatively high compared with groups lower in the hierarchy, while, compared with the other groups, first‐line managers evaluated OC as being much higher. Moreover, all self‐esteem factors were higher in employees who were in a managerial position than those who were not (*p* < 0.001).

**TABLE 4:** Mean values of factors of self‐esteem according to job title.

Factor	M (SD)	Mean values according to job title					
		Healthcare Professional	Specialist or other worker	Physician	First‐line manager	Leading upper management	*p* ^a^
Physical and emotional safety	3.397 (0.725)	3.268	3.482	3.719	3.838	4.072	<0.001
Collaboration and identity	3.345 (0.740)	3.232	3.423	3.580	3.689	3.796	<0.001
Objectives and creativity	3.137 (0.794)	3.019	3.185	3.331	3.710	3.137	<0.001

^a^ One‐way ANOVA/Kruskall–Wallis test.

There was no significant difference between age groups in terms of PE or OC, whereas a weak statistically significant difference was found between age groups for CI (*p* = 0.040). CI was highest in employees who were ≥60 years old (M: 3.511) and the lowest in those who were 30–39 years old (M: 3.246).

#### 4.2.3 Categories of human competencies and association with background variables

Among all categories of human competences, leadership was valued the lowest, with a mean score of 2.494 (SD: 0.951; Table 5). Healthcare professionals, other workers and physicians evaluated leadership as being quite low. The results also showed some disappointment towards upper management in lower hierarchy groups, while upper management evaluated their own actions to be better. The results showed that there is a need to improve the way upper management communicates, prepares employees for the future and shows care towards the well‐being of employees.

**TABLE 5:** Mean values of the categories of human competences according to job title.

Competence	*M* (SD)	Mean competence values according to job title					
		Healthcare professional	Specialist or other worker	Physician	First‐line manager	Leading upper management	*p* ^a^
Leadership	2.494 (0.951)	2.260	2.761	2.156	3.102	3.347	< 0.001
First‐line management	3.218 (1.072)	3.086	3.211	3.813	3.833	4.067	< 0.001
Culture	3.775 (0.815)	3.722	3.748	4.172	4.074	4.160	< 0.001
Skills	3.708 (0.732)	3.592	3.786	4.021	3.972	4.087	< 0.001
Processes	3.294 (0.842)	3.216	3.308	3.583	3.597	3.647	0.004

^a^ Kruskall–Wallis test.

First‐line management was at a moderate level compared with other competences (M: 3.218). According to the results, there is need to improve the leadership of first‐line managers to be more inspiring and encouraging. The actions of first‐line managers were evaluated as being more impartial and fairer in higher hierarchy groups than in healthcare professionals and other workers.

Culture (M: 3.775) and skills (M: 3.708) were the highest evaluated categories. All of the workers reported being confident that they were sufficiently skilled to perform their job effectively, but believed that there could be more opportunities for skill development. Regarding the working culture, there was found to be good level of interprofessional collaboration and a culture to help others. Moreover, the employees expressed feeling able to share their views in the work community.

Processes were scored as being moderate (M: 3.294) and were given the lowest scores by healthcare professionals (Table 5). There is a need to improve the ways in which the organization develops their operations and greater advantage could be taken of customer feedback and digitalization.

The results showed a statistically significant difference in leadership (*p* = 0.003) and processes (*p* = 0.007) between age groups. Leadership scored the highest in participants aged 50–59 years old (M: 2.646) and ≥ 60 years old (M: 2.662). The lowest mean score was 2.290 in participants aged 30–39 years old. Processes was also the highest in the oldest age group (M: 3.552) and the lowest in the 30–39 year group (M: 3.173). Employees in managerial positions gave higher scores to all competences compared to those who were not (*p* < 0.001).

## 5 DISCUSSION

### 5.1 Findings

The results show that the QWL is moderate in the employees of this public healthcare organization. The mean QWL index of the whole organization was 0.524, which is midrange of the QWL index. Similarly, the QWL has been found to be moderate among Chinese nurses (Wang et al., 2019, 2020) and low among nurses in Iran (Raeissi et al., 2019). The results of this study indicate that there is much room for improvement for the factors that contribute to QWL to increase the satisfaction level of employees. Investment in the development of the QWL is profitable, as the utilization level of the intangible employee assets is moderate. QWL is associated with organizational performance and productivity (Kesti et al., 2016; Pot & Koningsveld, 2009), occupational stress (de Lira et al., 2021) and burnout (Wang et al., 2019). QWL interventions can influence employee commitment under effective leadership styles (Nanjundeswaraswamy et al. 2020); thus, many benefits for the organizational performance could be gained through the development of QWL, where leaders are in a key role.

The QWL index was calculated with the outcomes of self‐esteem factors. Physical and emotional safety (FE) was the highest scored factor and represents a good foundation for QWL development. According to the theory basis of the QWL index, the FE should be high to fully utilize performance potential. However, the deviation of FE was also high, indicating that there are employees with problems in this area, particularly in healthcare professionals. Therefore, it would be beneficial to develop FE by aiming to decrease the deviation. Justice in management and decision‐making is one aspect of feelings of safety, which also influences job satisfaction and nurse retention (Zahednezhad et al., 2021). Some healthcare professionals, especially paramedical personnel, may encounter workplace violence (Sheikhbardsiri et al., 2022), which can decrease feelings of safety. Aminizadeh et al. (2022) found that job security is one important aspect in increasing QWL among paramedical personnel. The objectives and creativity factor had the lowest mean score; this factor relates to the positive attitude and enthusiasm of employees and has the greatest potential to increase QWL when it is developed (Kesti et al., 2016). Thus, there is much unused potential among employees, which, when taken into consideration, could have a positive effect on organizational outcomes. A transformational leadership style has been found to have a positive effect on organizational outcomes such as innovation and work climate (Cummings et al., 2018).

Leadership was scored low, indicating disappointment towards upper management. This suggests that there is much work to be done by upper management to develop QWL, including by developing their communication with subordinates. Previous studies have shown that human resource management has a significant role in developing better QWL (Wang et al., 2020). The results of our study also showed a considerable difference in the QWL index and other variables between those who were in a managerial position and those who were not. This indicates that the view of managers differs greatly from that of their subordinates. Consequently, to develop management and leadership, employees in a managerial position could take advantage of the views of their subordinates. Aminizadeh et al. (2022) found that to develop better QWL, it is beneficial to increase employee participation in decision‐making. Similarly, Raeissi et al. (2019) found that, among others, poor support from management and insufficient contribution to decision‐making were reasons for low QWL. Developing managerial competence can also increase workers' organizational commitment (Almutairi & Bahari, 2022.). Addressing staff shortages and improving competence in human resource management can also help to increase efficiency in work activities (Nobakht et al., 2018).

According to the results, the employees are fairly content with their working culture and skills. Hahtela et al. (2015) found that there is uncertainty towards the working culture among nurses in Finland; however, we observed no such unsatisfaction, although healthcare professionals and other workers evaluated culture as being lower compared with physicians and management. Moreover, the QWL was the lowest in healthcare professionals when compared to other professional groups. Age had no statistically significant effect on the QWL index in our study. Some previous studies have found older workers to have significantly higher scores in QWL (Raeissi et al., 2019), while others have found younger workers to have better QWL (Wang et al., 2019).

Healthcare services are suffering from widespread problems, including workforce shortages (WHO, 2020), burnout and job dissatisfaction (Scammell, 2016). The COVID‐19 pandemic has had a significant impact on the work tasks and content of employees and management of healthcare organizations (Braquehais et al., 2020; Varanka et al., 2022). Our results highlight that there is much that can be done through leadership to influence organizational outcomes, such as performance, job satisfaction and intentions to leave, which would contribute to easing the problems in the healthcare field.

### 5.2 Strength and limitations of the work

As a cross‐sectional study, our findings cannot offer information on causality. The research data were collected by the hospital district, so the researcher could not take part in the data collection. As the data were only collected from one organization, geographical differences may exist. The response rate was 40%, which is relatively good considering that response rates in online questionnaires tend to be low (Polit & Beck, 2017). During data collection, two follow‐up reminders were sent to respondents via e‐mail. Loss analysis was not possible, but it would have added information about the reliability of the study. However, as the number of respondents was quite large and given that public healthcare is highly regulated, public healthcare organizations share many similarities; therefore, our results are likely generalizable to Finnish healthcare organizations and other similar organizations.

Self‐reported questionnaires are associated with possible biases, given that there may be variability in the way in which questions are interpreted by respondents (Polit & Beck, 2017). Additionally, there exists a possible bias related to the time of measurement, in that the data were collected during the COVID‐19 pandemic, which has impacted healthcare organizations.

### 5.3 Recommendations for further research

In future research, it would be beneficial to study QWL interventions and their effects longitudinally, as well as to determine how QWL is associated with employee risks, such as sick absence and work disability. We also recommend further development of the QWL questionnaire and evaluation of the convergent and discriminant validity in the healthcare context.

## 6 CONCLUSIONS

Our findings show that the QWL in a public healthcare organization was moderate, with scope for improvement. There is a need to increase feelings of safety and to fully incorporate the potential of employees in terms of innovation and creativity. The practical implication of our study is that better QWL could be achieved by focussing on developing management and leadership competence, particularly by considering the views of employees and including them in decision‐making. Nurse managers should be aware that measuring QWL can increase knowledge‐based management. Theoretical implication is that QWL interventions to develop leadership skills and increase innovation and inclusion of employees are beneficial and the development of the QWL can positively impact many organizational outcomes. As healthcare professionals, largely nurses and midwives, were the most unsatisfied with the QWL, QWL interventions focussing on nurses and nursing management would likely serve to decrease differences between professional groups. We suggest that the effectiveness of QWL interventions focussing on nursing management should be studied further in future.

## CONFLICT OF INTEREST STATEMENT

The authors declare no conflicts of interest.

## Data Availability

The data has been reported in this study
